# General practitioners' beliefs about effectiveness and intentions to recommend smoking cessation services: qualitative and quantitative studies

**DOI:** 10.1186/1471-2296-8-39

**Published:** 2007-07-05

**Authors:** Florian Vogt, Sue Hall, Theresa M Marteau

**Affiliations:** 1 Health Psychology Section, Department of Psychology (at Guy's), Institute of Psychiatry, King's College London, London, UK; 2 Department of Palliative Care and Policy, School of Medicine at Guy's, King's College and St Thomas' Hospitals, King's College London, London, UK

## Abstract

**Background:**

General practitioners' (GPs) negative beliefs about smoking cessation services may act as barriers to them recommending such services to smokers motivated to stop smoking.

**Methods:**

In Study 1, 25 GPs from 16 practices across London were interviewed in this qualitative study. Framework analysis was used to identify key themes in GPs' beliefs about smoking cessation services. In Study 2, a convenience sample of 367 GPs completed an internet-based survey. Path-analysis was used to examine relationships between beliefs identified in Study 1 and intentions to recommend smoking cessation services.

**Results:**

In Study 1, GPs felt that smoking cessation assistance was best provided by others. GPs favoured local services (i.e. practice nurses offering stop smoking support) over central services (i.e. offered through the Primary Care Trust), mainly because these were seen as more personalised and accessible for patients. These beliefs appeared to influence GPs' beliefs about the effectiveness of services. In Study 2, GPs' beliefs had a large effect on their intentions to recommend both central services, (*f*^2 ^= .79) and local services, (*f*^2 ^= 1.04). GPs' beliefs about effectiveness and cost-effectiveness were key predictors their intentions to recommend central services and local services. Beliefs about the level of personalisation offered and smokers' likelihood of attending services had indirect effects on intentions to recommend services operating via beliefs about effectiveness.

**Conclusion:**

GPs vary in their perceptions of the effectiveness of smoking cessation services and their intentions to recommend these services vary in line with these beliefs. Interventions aimed at increasing the likelihood with which GPs recommend these services may therefore be more effective if they addressed these beliefs.

## Background

Helping people to stop smoking is one of the most effective ways of preventing premature death and reducing health inequalities [1]. In the UK, a main strategy for achieving this is to increase the number of smokers that use the National Health Service Stop Smoking Service. This service offers one-to-one or group support which is free at the point of delivery, and support with smoking cessation medications, nicotine replacement therapy (NRT) and bupropion (Zyban) [1]. These medications are available on prescription to all smokers who want to stop smoking independent of their desire to use formal support from the Stop Smoking Service [2]. Compared to the great majority of medical interventions [2,3], nicotine dependence medications and services, alone and in combination, are highly cost-effective. Fifteen percent of smokers who use the Stop Smoking Service do not smoke after 12 months [4] compared with 5% who quit on their own [5]. However, only 7% of smokers use the Stop Smoking Service [6].

As part of the Stop Smoking Service most Primary Care Trusts (PCTs) have a central service. The central services have been regarded as Level 3 service; support for smokers is offered by specially trained full-time staff, usually in groups. The central services also provide training for health professionals in the PCTs, mainly practice nurses and pharmacists, to provide what has been regarded as the Level 2 service. These training sessions usually involve a one-day session after which trainees can provide basic behavioural support and discuss nicotine dependence medications on a one-to-one basis at GP practices or pharmacies, respectively. The central services also offer booster sessions for these local advisors, offering continued support and training. Despite the high level of use by smokers of Level 2 services current evidence from the Stop Smoking Service suggests that group support is more effective than one-to-one support [7,8]. The reasons for this difference are not well understood but they may include more experienced advisors at the central services, the benefits of group support, and more time being spent with advisors [8].

Smoking cessation guidelines [2] and the new NICE Public Health Intervention Guidance [9] recommend that general practitioners (GPs) advise all smokers to stop smoking and provide nicotine dependence medications and/or refer smokers who are motivated to stop smoking to the Stop Smoking Service. It is estimated, however, that advice to quit is given in only 20%-30% of UK primary care consultations with smokers [10]. Furthermore, one study estimated that just 6% of GPs had referred smokers to the central services, and only 41% had referred smokers to nurses trained in smoking cessation in the previous month [11]. Another study showed that only 5% of smokers were advised about NRT by their GP [12].

Failure to implement evidence-based guidelines is not restricted to smoking cessation [13,14]. Interventions to increase adherence to guidelines using a wide-variety of methods including incentives, prompts/reminders, and education have had mixed results and there is no clear evidence to favour any particular strategy [14]. Critiques of this large literature highlight that most interventions lack an explicit rationale or theoretical basis [13,15]. A first step to developing an intervention to increase the frequency with which GPs refer smokers who are motivated to the Stop Smoking Service is to identify the factors that may influence such referrals. Motivational theories propose that motivation to perform a behaviour is a proximal determinant of the performing the behaviour [16-19]. Interventions should therefore target factors that determine motivation. Examples of such determinants include beliefs about the consequences of behaviour and attitudes towards performing the behaviour.

A systematic review showed that while the majority of GPs and family physicians do not hold negative beliefs and attitudes towards discussing smoking cessation with their patients, a sizeable minority do [20]. Forty-two percent believed that discussing smoking cessation was too time consuming, 38% believed it was ineffective, and just over a fifth (22%) reported lacking confidence in their ability to discuss smoking cessation with their patients. Few studies, however, have assessed beliefs about smoking cessation services. One that did reported that around 20% of GPs believed that directing smokers to the central services was inappropriate and 9% thought that directing smokers to such services was ineffective [21]. No studies were found of GPs' beliefs about local services offered by practice nurses or other health professionals. Therefore, little is known about GPs perceptions of smoking cessation services. The quantitative nature of the available evidence furthermore limits understanding of GPs' perceptions of smoking cessation services. For example, whilst this research showed that many GPs believed that assisting smokers at central services was inappropriate, why they believed this is not known. Identifying the specific beliefs that underlie some of the broad categories of beliefs is likely to be useful for designing interventions to change GPs' beliefs.

A major feature of qualitative methods is their ability to describe and display phenomena as experienced by the study population in fine detail and in the study participants' own terms. It therefore offers the opportunity to 'unpack' phenomena, to see what they are about or what lies behind them [22]. Qualitative research is therefore well positioned to map the range of GPs' beliefs about smoking cessation services. Qualitative research also allows associations that occur in people's thinking or acting to be identified [22] . Using qualitative methods, the aim of Study 1 is to provide an in-depth understanding of the basis for GPs' beliefs about smoking cessation services. This is followed by a second study aimed at describing the prevalence of these beliefs and the strength of their association with intentions to recommend smoking cessation services.

## Study 1: a qualitative study of GPs' beliefs

### Methods

#### Design

Cross-sectional individual interviews with GPs.

#### Participants

Twenty-five GPs, whose practices are part of the Medical Research Council General Practice Research Framework (MRC GPRF), were interviewed. Ten of the GPs were female and fifteen were male (age range 27 to 60).

#### The interview

Interviews were conducted by a researcher trained in qualitative interview techniques (FV). A semi-structured interview schedule was used covering topics relating to discussing smoking with smokers, NRT, bupropion, and support offered at the central services and local services. Emphasis was given to the criteria used in deciding whether or not treatments are introduced into health care [23], that is, their effectiveness and cost-effectiveness. The interview schedule was piloted with three GPs from a London general practice, and refined where appropriate. Interviews were audio-taped and transcribed verbatim.

#### Data analysis

The data were managed using NVivo software for qualitative data analysis and analysed using the framework method [22]. Framework analysis has five stages: familiarisation, identifying a thematic framework, indexing, charting, and mapping and interpretation. Details of the analysis process are shown in Table 1. Internal validity was established through the 'constant comparative method' [24], involving constant and repeated checking of the interpretation of the data, which is inherent to the five stages of framework analysis [22]. In addition, the thematic analysis was supported and verified by two experienced researchers by ascertaining consensus in the interpretation. The internal validity was enhanced by displaying quotations to supplement the analysis where quotations explicitly documented linkages or explanations. To address external validity, 'methods triangulation' [22], which relies on generating data by another method, was used. Study 2 serves for triangulation and validation of the themes that emerged from Study 1. Only themes regarding smoking cessation services are reported. Themes relating to smoking cessation medications are reported elsewhere [25].

**Table 1:** Process of data analysis

FV reads interview transcripts and generates codes for beliefs reported by GPs about intervening with smokers and about smoking cessation medication and services.
FV reads and compares the codes to identify themes within these: the principal beliefs GPs reported about intervening with smokers and about smoking cessation medication and services.
Data relating to each theme are assembled. FV, SH, and TMM independently read these and discuss definitions of themes and the data within these. During this process some definitions are altered and some data re-coded.
FV re-codes all transcripts for these themes.
FV begins coding for sub-themes within themes: more detailed variations in GPs' thinking within themes. Sub-themes include GPs' explanations for reasons behind preferring local services to central services.
The process of coding for sub-themes includes building a framework containing themes and sub-themes. This process includes shifting themes to sub-themes and vice versa.
The framework is arranged in tables using text segments to represent the themes in order to facilitate understanding of the data
FV, SH, and TMM study the tables to gain an understanding of themes and sub-themes and decide on main issues. Disagreements are discussed and amendments made where appropriate.

#### Procedure

The MRC GPRF sent invitation letters to all 128 practices in the framework in the Greater London area. Of these, 16 agreed to take part in the study. From these, 30 GPs agreed to be interviewed and gave written consent to take part in the study. Five GPs did not have time to be interviewed in the limited data collection phase of the study. GPs were interviewed in their practices. At the start of the interview they were assured of anonymity. The interviews lasted between 10 and 30 minutes, depending on how much each participant had to say. After the interviews, all participants were offered €20 book tokens as compensation for their time.

## Results

### Referring motivated smokers

A central theme that emerged from the interviews was that GPs believed that their role was primarily to identify smokers and advise them to stop smoking, but not to provide more intensive support for smoking cessation. Advice included pointing out the dangers of smoking as well as giving a clear recommendation to stop. However, GPs felt behavioural support should be provided by others and therefore referred smokers who were motivated to stop.

'... my job obviously is to ask people if they are smoking and I document how much they are smoking and advise them to stop and then I tell them about the systems that are in place to help them ... (GP3)'.

Beliefs underlying this decision included not having sufficient time to support smokers. Another belief was that GPs perceived other health professionals as better suited to provide intensive behavioural support because these were seen as having more expertise and experience in providing such support. GPs clearly appreciated the availability of services to which they could refer smokers for intensive support.

'I think it's a good use of my time to raise the issue and to give them [smokers] encouragement. I don't think it's the best use of my time because others do it better and I haven't got enough time to do the smoking cessation work as it were (GP15)'.

### Preference for in-house support over PCTs' central services

GPs were aware of different options when deciding where to refer motivated smokers to intensive support. These included PCTs' central service, in-house support provided by a practice nurse, and local pharmacists offering smoking cessation support. Although GPs were aware of alternative services, they preferred to use the in-house support if this was available.

'I know that there is a PCT-based service that we can refer on to, but we do have our nurse here who does smoking cessation clinics, so anybody that I see that wants to stop smoking I tend to get to see our practice nurse (GP21)'.

When discussing the effectiveness of central service and in-house support, GPs agreed that both helped smokers to stop smoking even though many smokers who used either of the services would not succeed in stopping smoking. GPs presumed that smokers would receive intensive support and counselling at the central service and their staff were regarded as experts in the field possessing the most up to date evidence of smoking cessation methods. Contributing to this positive perception was the belief that a dedicated central service would have sufficient time to assist smokers.

'I think specialist clinics are a good idea. ... they're doing it full time and so they're aware of what's around (GP10)'.

GPs reported that practice nurses who provided in-house support often provided feedback to GPs that the service helped smokers. Contributing to a sense of effectiveness was GPs' perception that practice nurses had received the relevant training and hence possessed the necessary expertise. This included being able to support smokers in using pharmacological treatments for nicotine dependence and monitoring smokers' nicotine consumption levels by using carbon dioxide monitors.

'I will often refer them to the nursing staff who actually run clinics, and I think they've been to courses where I think it's Level B smoking advice they provide, and they'll sort of monitor the patient with the carbon monoxide monitors, and I think that is extremely useful for most of our patients (GP7)'.

Whilst both central services and in-house support were seen as offering expertise, two features of in-house support seemed to contribute to GPs' preference for this over the central services. One perceived difference between central services and in-house support was that the support practice nurses offered was more personalised than that provided by central services, which they believed made it more effective. The perception that the in-house support was more personalised was supported by the recognition that smokers and staff were familiar with each other and that smokers knew the setting.

'We're a very small practice, we take a lot of individual interest in our patients, and I think that has helped them to stop smoking more than sending them off in a rather nebulous way to a place where they learn en masse to stop smoking (GP5)'.

The other feature was that in-house support was seen as easier to access than central services. This was seen as having several implications, including a positive impact on the effectiveness of in-house support. One aspect about the ease of access GPs valued was that the in-house support was very responsive, capitalising on smokers' high levels of motivation. By contrast, some GPs expected that the central services would have a lengthy waiting-list, prohibiting smokers from receiving support at the time when they were ready to stop smoking.

'We occasionally refer to external smoking cessation clinics at other sites, but generally it's something that occurs within the practice. I think there's a huge benefit to people who look at stopping smoking. And I think it also works a little bit better if we make it, our time, available, because people often respond when they are ready to stop, and if they actually make a decision that they're ready to stop, if they can access something relatively promptly that tends to work a little bit better than having to wait some length of time before they access a service of that sort (GP20)'.

A second aspect about the ease of access was that GPs also believed that smokers could enrol with the in-house support with minimal effort. Smokers, GPs said, could simply make an appointment with the practice nurse when they were at the practice. A third aspect about the ease of access was convenience of locality. In comparison to in-house support, central services were seen as difficult to get to. This was seen as having a negative influence on the effectiveness of the central services because it was expected to lead to greater drop-out.

'I mean I suspect there's a higher risk of DNA [Do Not Attend], higher risk of people not turning up, higher risk of people falling out if they're having to travel further to access such services (GP9)'.

Apart from being poised for increased drop-out, inconvenience of location was also seen as decreasing the potential of the central services on smoking rates in the population because it would deter smokers from enrolling. GPs perceived this as a particular issue for the elderly and people without transport.

'I think it's very good [in-house support], because I think patients like to have services that are local to them, and I think they are much more likely to attend a service within their own GP surgery... (GP21)'.

GPs also valued the in-house support for promoting continued contact with patients and a comprehensive approach to patients' health.

'I think it is very important that the patient is having that aspect of their health looked after at the GP surgery, who is looking after a lot of other aspects of their health. And to me that is a strength, and maybe a disadvantage of the specialised centres... (GP14)'.

Supporting patient preference for familiar and personalized services was seen as another advantage of the in-house support. GPs believed that smokers preferred the in-house support because they were familiar with the practice and the staff, and because smokers preferred a more personal one-to-one approach.

'Well I think it's [in-house support] a very good service. It's well regarded by us as a practice, patients are attracted to it, we promote it, ... I think many patients value the one-to-one approach rather than a group approach (GP15)'.

When discussing whether in-house support and central services were effective enough to justify their costs it appeared that GPs believed that both were cost-effective. GPs based these evaluations on the effectiveness of the services at reducing the prevalence of smoking and the incurred future cost-savings that would result from the reduction of smoking related morbidity.

'I think even if you make just a few people give up smoking, you're still saving the NHS a massive amount of money from the morbidity and the mortality of what smoking does [in-house support] (GP12).' 'And what about NHS clinics [central services]?' (FV). 'The same thing, it must be saving money in the long term (GP12)'.

GPs also believed that offering support for smokers in groups, as practiced at the central services, would have a beneficial impact on their cost-effectiveness because it allows few health professionals to support many smokers, resulting in low salary costs per supported smoker. Factors perceived as limiting the cost-effectiveness of central services included, firstly, a perception that such services would see a high rate of drop out, and secondly, high maintenance costs. Maintenance costs incurred included salary for staff, who are specifically employed and trained to provide smoking cessation support, and costs associated with providing the premises at which the service would be offered.

'Well I wouldn't think they're that cost-effective [central services], because at the end of the day the Government's employing extra people, and also obviously added costs to that for rent of the place and whatever, the tools they use, when it's something that has been done in the surgeries, which had no extra costs to the PCTs, but maybe to the GPs because of the extra time the nurse had to put into it (GP8)'.

The high costs of paying the practice nurses for the additional service of providing smoking cessation support in-house was noted as a factor that reduced the cost-benefit of offering such a service in-house. In particular it was noted that the costs of such an in-house support provided at their practice would directly affect their budget, whereas the costs of the central services would be paid for by the PCT. On the other hand, the existence of sites in which the in-house support were provided by way of the GP surgeries was mentioned as a factor in support of the cost-effectiveness of the in-house support.

'How cost-effective do you think this service [in-house support] is (FV)?'. 'I would say moderately cost-effective, mainly because now the nurse time is quite expensive, mainly for that. But previously I think it would have been a cost-effective clinic to run. But now because her time is a lot more expensive and you have to reduce hours and it's not really making you any money, so again that's going to reflect (GP9)'.

## Results summary

GPs believe that while advising smokers to stop smoking is a part of their role, providing intensive smoking cessation support is not. GPs prefer to refer smokers to their practice nurse if possible, rather than using central services. Underlying this is a preference for services that offer an accessible and personalised service.

## Study 2: a quantitative study of GPs' beliefs

The aims of Study 2 are: (i) to estimate the prevalence of the beliefs identified in Study 1, and (ii) to describe the strength of relationships between beliefs and intentions to recommend services to patients.

### Methods

#### Design

A cross-sectional self-administered questionnaire based online survey.

#### Participants

Three-hundred and sixty-seven general practitioners completed the survey. All were users of an internet-based medical information service provider. At the time of study, 4743 UK GPs (~12% of UK GPs) were registered users of this information services provider. Of the 381 GPs who were invited to participate, fourteen (4%) did not complete the questionnaire. Seven (2%) declined and seven (2%) deferred completion. Respondents were generally representative of the general population of English GPs, based on the Department of Health Statistics for General Medical Practitioners, with a bias towards men responding (about 60% of GPs in the UK are male) (Table 2). This bias reflects the profile of GPs that are registered with the service provider.

**Table 2:** Demographic and background details of participants

**Characteristic**	**Levels**	**N**	**%**
Gender	Female	60	16.3%
	Male	307	83.7%
Decade of qualification	1960s	11	3%
	1970s	122	33.2%
	1980s	146	39.8%
	1990s	86	23.4%
	2000s	2	0.5%
Commitment	Full time	323	88%
	Pat time	44	12%
Country of training	UK	327	89.1%
	Other	40	10.9%
Geographical location	London	32	8.7%
	South East	63	17.2%
	South West	40	10.9%
	West Midlands	41	11.2%
	Eastern	46	12.5%
	Trent	44	12%
	North West	56	15.3%
	Northern and Yorkshire	45	12.3%
Presence of trained practice nurse	No	89	24.3%
	Yes	277	75.5%
	missing	1	0.3%

#### Procedure

A questionnaire was presented to any member of the service provider registered as a general practitioner upon accessing the service during four days in November 2004. When members log on to visit the Web site, they must give their General Medical Council number. Software checks this information as well as a list of available questionnaires. Members then have three choices: (i) complete the questionnaire immediately, (ii) defer completion to another time, or (iii) refuse completion. The service provider carries out regular profit and not-for-profit survey research among its members and has been successfully used for academic purposes [26]. Members provide consent to take part in research studies when they join the service. Rewards are offered to members if they respond to questionnaires on a regular basis.

#### Measures

The questionnaire content was designed on the basis of the results from Study 1. It was piloted with 22 GPs using the same method as the main study. On the questionnaire it was explained that local services offered by practice nurses or other local health professionals would provide one-to-one support, and that the PCTs' central services offered support in groups.

##### Intention

Intention is defined as the expressed motivation to perform some behaviour or achieve some goal [27]. Intentions about the next month were measured using the stems "...do you intend to recommend to all motivated smokers ..." and "...how likely is it that you will recommend to all motivated smokers..." with regards to the central services (Cronbach's α =.917) and the local services (Cronbach's α =.927). The "... intend to ..." stem had a response range from 1 (definitely do not) to 7 (definitely do). The "... how likely ..." stem had a response range from 1 (very unlikely) to 7 (very likely). The mean of the two items was determined to form an intention one scale for central services and one for local services.

##### Beliefs

Perceived effectiveness was measured using the stem "... is effective at helping motivated smokers to stop smoking". Perceived cost-effectiveness was measured using the stem "...is effective enough to justify its cost" [11]. Perceived sufficient personalisation of services was measured using the stem "...provide motivated smokers with a service that is sufficiently personalized". Perceived low-attendance to services was measured using the stem "few motivated smokers will go to...". All items were measured with regards to the PCTs' central services providing group support and local services providing one-to-one support. Beliefs were measured using a response range from 1 (strongly disagree) to 7 (strongly agree).

#### Path analysis

Path analysis is a concise way to organise causal thinking and is an extension of the statistical method of multiple linear regression [28]. In addition to multiple regression analysis, path analysis assembles the antecedents of the outcome variable into a structure of presumed causal relationships. Given such a presupposed causal model, path analysis estimates the magnitude of the linkages between the variables. Causal pathways operating via two or more variables indicate indirect effects. Indirect effects are very similar to mediated effects. A mediator is a variable *"to the extent that it accounts for the relation between the predictor and the criterion" *[[29], p. 1176]. To establish the significance of indirect effects operating through two or more variables covariance structure software is considered superior [30]. The covariance structure software AMOS [31] was used to calculate the estimate path coefficients using a bootstrapping method (2000 bootstrap samples were used) [32]. The path models were based on Study 1. All beliefs were initially considered as direct predictors of intentions (non significant paths were removed from the models). The Squared Multiple Correlation (SMC) of each outcome variable was calculated. The SMC is the equivalent to an R-squared (R^2^) in linear regression [33]. Effect sizes were calculated for the variance explained in intentions using *f*^2 ^= R^2^/(1-R^2^) [34]. According to Cohen an *f*^2 ^value of 0.02 is small, an *f*^2 ^value of 0.15 is medium, and an *f*^2 ^value of 0.35 is large.

#### Data preparation

Prior to analysis, the variables were examined for accuracy of data entry, missing values, and violations of multivariate assumptions and passed all required tests (some variables were not normally distributed but bootstrapping is unaffected by this). There were few missing data, well below 5% in all instances. Missing values were replaced with the mean responses of the variable.

## Results

### Prevalence of beliefs and intentions

Sixty-six percent of GPs agreed that central services were effective, while 82% agreed that local services were effective (Table 3). Fewer than 50% agreed that central services and local services were cost-effective. Twenty-nine percent of GPs did not intend to recommend central services to smokers wishing to stop smoking. Forty percent did not intend to recommend local services to smokers.

**Table 3:** Prevalence of beliefs and intentions

	Proportion			
Beliefs	**Agree (>4)**	**Neutral (4)**	**Disagree (<4)**	**Missing**
Central services are effective.	66.0% (241)	22.5% (82)	11.5% (42)	0.5% (2)
Central services are cost-effective.	48.5% (177)	29.9% (109)	21.6% (79)	0.5% (2)
Local services are effective.	81.6% (297)	11.8% (43)	6.5% (24)	0.8% (3)
Local services are cost-effective.	47.7% (175)	21.0% (77)	31.4% (115)	-
Intentions	**Intend (>4)**	**Neutral (4)**	**Do not intend (<4)**	**Missing**

Intention to recommend central services.	63.8% (234)	7.1% (26)	29.2% (107)	-
Intention to recommend local services.	51.2% (188)	8.4% (31)	40.3% (148)	-

### Path analysis for intentions to recommend central services

**Figure 1:**
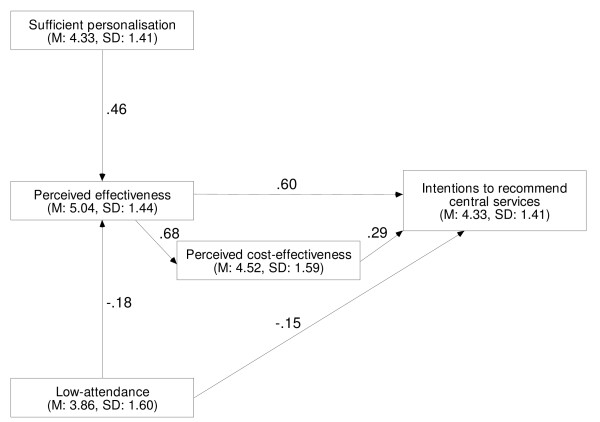
Central services path model; unstandardised coefficients, M = mean, SD = standard deviation.

'Perceived effectiveness of central services', 'perceived cost-effectiveness of central services', and 'perceived low-attendance at central services' had direct effects on 'intentions to recommend central services' (Table 4, Figure 1). 'Perceived effectiveness of central services' also had a direct effect on 'perceived cost-effectiveness of central services'. 'Perceived sufficient personalisation of central services' and 'perceived low-attendance at central services' had direct effects on 'perceived effectiveness of central services'. 'Perceived effectiveness of central services' had an indirect effect on 'intentions to recommend central services' operating through 'perceived cost-effectiveness of central services'. 'Perceived sufficient personalisation of central services' and 'perceived low-attendance at central services' had indirect effects on 'intentions to recommend central services' operating through 'perceived effectiveness of central services'. Adding up the direct and indirect effects, 'perceived effectiveness of central services' (B = .79) had the largest total effect on 'intentions to recommend central services', followed by 'perceived sufficient personalisation of central services' (B = .36), 'perceived low-attendance at central services' (B = -.30), and 'perceived cost-effectiveness of central services' (B = .29).

**Table 4:** Central services: direct, indirect, and total effects on beliefs and intention

**Dependent variable**	**Independent variable**	**Effects**^a^		
		**Direct**	**Indirect**	**Total**
Intentions to recommend central services SMC = 0.44, 95% CI: 0.36 to 0.54, p < 0.01, *f*^2 ^= 0.79.	Perceived effectiveness	.60***	.20***	.79***
	Perceived cost-effectiveness	.29***	-	.29***
	Sufficient personalisation	-	.36***	.36***
	Low-attendance	-.15**	-.15***	-.30***
Perceived cost-effectiveness SMC = 0.37, 95% CI: 0.28 to 0.47, p < 0.01.	Perceived effectiveness	.68***	-	.68***
	Sufficient personalisation	-	.31***	.31***
	Low-attendance	-	-.13***	-.13***
Perceived effectiveness SMC = 0.32, 95% CI: 0.24 to 0.41, p < 0.01.	Sufficient personalisation	.46***	-	.46***
	Low-attendance	-.18***	-	-.18***

^a ^unstandardised coefficients, **p < 0.05, ***p < 0.01.

### Path analysis for intentions to recommend local services

**Figure 2:**
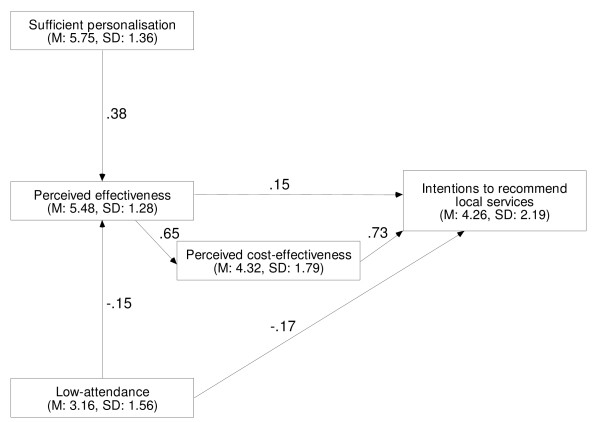
Local services path model; unstandardised coefficients, M = mean, SD = standard deviation.

'Perceived effectiveness of local services', 'perceived cost-effectiveness of local services', and 'perceived low-attendance at local services' had direct effects on 'intentions to recommend local services' (Table 5, Figure 2). 'Perceived effectiveness of local services' also had a direct effect on 'perceived cost-effectiveness of local services'. 'Perceived sufficient personalisation of local services' and 'perceived low-attendance at local services' had direct effects on 'perceived effectiveness of local services'. 'Perceived effectiveness of local services' had an indirect effect on 'intentions to recommend local services' operating through 'perceived cost-effectiveness of local services'. 'Perceived sufficient personalisation of local services' and 'perceived low-attendance of local services' had indirect effects on 'intentions to recommend local services' operating through 'perceived effectiveness of local services'. Adding up the direct and indirect effects, 'perceived cost-effectiveness of local services' (B = .73) had the largest total effect on 'intentions to recommend local services', followed by 'perceived effectiveness of local services' (B = .63), 'perceived low-attendance at local services' (B = -.26) and 'perceived sufficient personalisation of local services' (B = .24).

**Table 5:** Local services: direct, indirect, and total effects on beliefs and intention

Dependent variable	Independent variable	**Effects**^a^		
		**Direct**	**Indirect**	**Total**
Intentions to recommend local services SMC = 0.51, 95% CI: 0.43 to 0.58, p < 0.01, *f*^2 ^= 1.04.	Perceived cost-effectiveness	.73***	-	.73***
	Perceived effectiveness	.15**	.48***	.63***
	Sufficient personalisation	-	.24***	.24***
	Low-attendance	-.17**	-.09***	-.26***
Perceived cost-effectiveness SMC = 0.22, 95% CI: 0.14 to 0.31, p < 0.01.	Perceived effectiveness	.65***	-	.65***
	Sufficient personalisation	-	.25***	.25***
	Low-attendance	-	-.10***	-.10***
Perceived effectiveness SMC = 0.26, 95% CI: 0.17 to 0.35, p < 0.01.	Sufficient personalisation	.38***	-	.38***
	Low-attendance	-.15***	-	-.15***

^a ^unstandardised coefficients, **p < 0.05, ***p < 0.01.

## Discussion

Study 2 showed that while the majority of GPs perceived smoking cessation services as effective, some did not. GPs were divided about whether smoking cessation services were cost effective. Path analysis showed that beliefs about the effectiveness and cost-effectiveness of smoking cessation services had strong effects on their intentions to recommend these services to motivated smokers. Path analysis supported the findings from Study 1 by showing that beliefs about low-attendance at smoking cessation services and the personalisation of smoking cessation services had effects on intentions to recommend smoking cessation services working via beliefs about the effectiveness.

### Beliefs about assisting smokers to stop smoking

According to Study 1 GPs prefer not to assist smokers in stopping smoking, preferring to refer them to other health professionals. This finding is generally mirrored by smoking cessation guidelines. A concern remains, however, regarding the quality and effectiveness of this transfer of responsibility for helping smokers to stop smoking. Many smokers fail to attend for more intensive support, even after initially agreeing to do so [35,36]. A consequence of this may be that smokers try to quit unaided if their GP does not try to assist them.

### Preference for in-house assistance

Study 1 also suggested that GPs preferred to refer smokers to practice nurses for behavioural support. This is supported by survey findings showing that only 6% of GPs made referrals to central services whilst 41% reported referring smokers to practice nurses in their own practices for help with smoking cessation [11]. In contrast, Study 2 suggested that GPs were at least equally motivated to recommend smokers to use central services as local services. One explanation for the divergent findings may be that the intention measure used in Study 2 may capture GPs' intentions to recommend both of these services in general, but fails to distinguish that GPs may plan to recommend local services before recommending central services. Although patients may prefer local services, GPs may want to consider recent evidence from the Stop Smoking Service that suggests groups may be more effective (McEwen 2000; Judge 2006).

### Beliefs about effectiveness

The proportion of GPs that perceived central services as ineffective in Study 2 confirms findings of previous studies conducted among English and Welsh GPs [21]. It extends them by exploring so far undocumented beliefs about the effectiveness of local services. However, a question that remains is how GPs interpret effectiveness. They may interpret it (i) with reference to whether smokers are helped at all, or (ii) with reference to the clinical impact provided (e.g. low Number Needed to Treat (NNT) [37]). Whilst a negative response to the former can be interpreted as caused by lack of knowledge or rejection of the evidence, the latter interpretation may reflect GPs' evaluations of the perceived clinical impact informed either by personal experience, evidence or both. Given the large proportion of smokers that will fail despite using the most effective treatments, such assessments of clinical impact may be considered valid. Future research needs to address how GPs interpret effectiveness. There is no official guidance on a recommended level of NNT and meta-analyses provide no guidance on the value of the clinical impact of smoking cessation medications and services. Nevertheless, combined treatment (including behavioural support and medications) produced a NNT of 50 to save one life-year [38]. This compares favourably with other medical interventions, such as, Statins in primary prevention (NNT = 107) [39].

In the current study the more that GPs believed smoking cessation services were ineffective the less likely they were to report intending to recommend them. If GP' beliefs about the effectiveness of smoking cessation services are important, as is consistent with these findings, what factors influence their formation? In Study 1 some beliefs were identified that appeared to be underlying GPs' preference for smoking support offered by practice nurses. One was the belief that the central services would not be sufficiently personalised and the other was that they were difficult to access. Study 2 showed that GPs' beliefs about whether smokers would attend central services and local services and whether these two services were sufficiently personalised influenced GPs' intentions to recommend central services and local services via beliefs about the effectiveness of central services and local services. Thus, these two beliefs may be important targets when designing interventions to increase GPs' beliefs about the effectiveness of central services and local services.

### Beliefs about cost-effectiveness

Fewer than half the GPs surveyed believed that central services and local services were cost-effective. Beliefs about cost-effectiveness were directly related to intentions to recommend smoking cessation services, and this relationship was particularly strong for local services. These findings, illustrating the potential importance of beliefs about cost-effectiveness, contribute to the findings from a recent study showing that GPs who believed that NRT or bupropion were effective enough for the NHS pay for them, also believed that NRT and bupropion should be available on prescription [40]. The current study is the first to illustrate the importance of GPs' beliefs about cost-effectiveness with regards to GPs making smoking cessation services available for smokers. Perceptions of the cost-effectiveness of local services were probably a result of local services being seen as very effective (82% agreed that it was effective) and fairly expensive. The strong association between beliefs about cost-effectiveness and intentions to recommend local services may be a consequence of GPs being more influenced by expense than benefit once expense rises above a certain level. This reflects the well described observation that people in general are more influenced by preventing loss than by achieving gain [41]. Alternatively, the expense of local services may exert a strong influence because their costs are particularly salient: GPs directly bear the cost of in-house support whilst the costs of central services is realized across the NHS. More salient beliefs appear to exert stronger influences on intention and behaviour than non-salient beliefs [42]. In summary, GPs appear to consider cost in relation to effectiveness, in keeping with the recommendation that they do so by agencies such as the NICE [23].  Unfortunately, however, GPs' judgments do not always match the accepted evidence, with many judging some of the most cost-effective of all health care interventions, that is, smoking cessation services, as not cost-effective.

### Strengths and limitations

#### Study 1

A strength of this study is that qualitative methods allowed detailed description of previously unrecorded beliefs. However, financial and time constraints limited the length of the interviews, thus limiting the depth with which beliefs could be explored. Furthermore, the sample of the current study was restricted to GPs working in MRC GPRF-registered practices. Although MRC GPRF practices are representative in terms of the distribution of partnership size compared to the distribution of practices in the UK, these practices volunteer to dedicate some of their efforts to enhancing evidence-based medicine. GPs working in such practices and those that participated in the current study are thus likely to have a more favourable view of evidence-based medicine than GPs working in other practices. In addition, only a proportion of GPs from GPRF practices volunteered making the sample potentially unrepresentative of GPRF practices on the whole. It is suspected that those who volunteered may have been more favourable on smoking cessation medications and services than those GPs who did not.

#### Study 2

The predictive ability of the models used to explain intentions to recommend smoking cessation services compares satisfactorily to the effects sizes achieved in predicting intentions that are generally reported with motivational theories (e.g. Theory of Planned Behaviour) [43]. In addition, effect sizes in the current study are based upon specific beliefs instead of more abstract and general concepts such as attitudes included in the Theory of Planned Behaviour. By using specific beliefs the current studies suggest specific beliefs to target to increase GP referral to central services and local services. There are several limitations. Due to financial limitations only a small number of items could be included in the survey. This limited (i) the range of beliefs that could be assessed (e.g. perceived cost, beliefs about the consequences of behaviour), and (ii) the number of items used to assess beliefs. The latter prohibited removing measurement error and thereby decreased the power of the analysis [43]. The presumed causal sequences were tested and not refuted although path analysis can not ultimately prove their validity. Finally, the sample was not randomly selected from the population of English GPs and although the sample broadly reflected demographic characteristics of GPs' in the UK, GPs responding via the Internet might be unrepresentative in other ways that biases the results obtained. GPs responding via the internet may be more or less favourable towards smoking cessation services than the wider population of GPs. On the current topic evidence for the generalisability of results is provided by the observation that beliefs about the effectiveness of central services were similar to those found in a randomly selected sample of GPs [21]. The current sample had a male bias when compared with the national population of GPs, reflecting the profile of GPs that are registered with the service provider. However beliefs and intentions of male and female GPs did not differ in the current study (analysis not shown).

## Conclusion

Study 1 identified several reservations amongst GPs about the benefits of smoking cessation services. Study 2 provided evidence that these beliefs are prevalent and showed that they had large effects in predicting GPs' intentions to recommend smoking cessation services to smokers. Path analysis substantiated the presence of specific beliefs underlying perceptions of effectiveness and cost-effectiveness of smoking cessation services, the two key predictors of GPs' motivation to recommend patients to use these services. Addressing these beliefs may be important in encouraging GPs to refer more smokers to use these services.

## Competing interests

The author(s) declare that they have no competing interests.

## Authors' contributions

FV, SH, and TMM are responsible for the design of the studies; FV performed the interviews; FV was responsible for the acquisition and analysis of the data; SH and TMM participated in the analysis and interpretation of results; FV, SH, and TMM contributed to the conception and design of the paper; FV drafted the paper; SH and TMM critically revised the paper. All authors read and approved the final version of manuscript.

## Pre-publication history

The pre-publication history for this paper can be accessed here:


